# Physiology-Guided Coronary Revascularization Versus Angiography in Chronic Coronary Syndromes: A Systematic Review of Major Adverse Cardiovascular Events

**DOI:** 10.7759/cureus.102008

**Published:** 2026-01-21

**Authors:** Melina Carlos, María Verónica López Miño, Lincoln Xavier Naranjo Palacio, John Manuel Dorado Ramírez, Andrea Cecilia Lara Grados, Sergio Daniel Zabaleta Orozco, Martín José Saquicela Vasquez, Iván Marcelo Rhor Rivadeneira

**Affiliations:** 1 Internal Medicine, Instituto Mexicano del Seguro Social, Torreon, MEX; 2 Cardio-oncology, Carlos Andrade Marín Hospital, Quito, ECU; 3 Nephrology, Valley Kidney Care, McAllen, USA; 4 Emergency/General Practice, Clinica Internacional de Traumatología, Quito, ECU; 5 Internal Medicine, Instituto Mexicano del Seguro Social, Mexico City, MEX; 6 Medicine Department, Ministry of Public Health, Quito, ECU; 7 General Practice, Universidad Surcolombiana, Neiva, COL; 8 Medicine Department, Universidad Catolica Santiago de Guayaquil, Guayaquil, ECU; 9 Medicine Department, Ministerio de Salud Publica, Guayaquil, ECU

**Keywords:** angiography-guided revascularization, chronic coronary syndromes, fractional flow reserve, percutaneous coronary intervention, physiology-guided revascularization

## Abstract

Coronary angiography often fails to reflect the physiological significance of coronary stenoses in patients with chronic coronary syndromes (CCS), defined according to the contemporary European Society of Cardiology (ESC) criteria as stable coronary artery disease (CAD) without acute coronary syndrome. This review aims to compare physiology-guided versus angiography-guided coronary revascularization across percutaneous and surgical strategies in stable CAD. This systematic review followed the Preferred Reporting Items for Systematic Reviews and Meta-Analyses (PRISMA) 2020 guidelines and included randomized controlled trials and observational studies comparing physiology-guided (fractional flow reserve (FFR), instantaneous wave-free ratio (iFR), quantitative flow ratio (QFR)) and angiography-guided revascularization in CCS. PubMed, ScienceDirect, and Cochrane Library were searched. Risk of bias was assessed using the Cochrane Risk of Bias tool and Newcastle-Ottawa Scale, with narrative synthesis applied due to heterogeneity. Physiology-guided coronary revascularization was associated with lower early major adverse cardiovascular events (MACE) after percutaneous coronary intervention (PCI), with similar long-term outcomes compared with angiography-guided strategies. In the FAME trials, FFR-guided PCI reduced one-year MACE, while differences attenuated at two and five years. Across surgical and mixed revascularization studies, composite ischemic outcomes were largely comparable between physiology-guided and angiography-guided approaches. In FAME 3, FFR-guided PCI was compared directly with coronary artery bypass grafting (CABG), demonstrating no significant difference in death, stroke, or myocardial infarction at five years, although myocardial infarction and repeat revascularization were more frequent with PCI, reflecting differences between revascularization modalities rather than physiology-guided versus angiography-guided decision-making. Mortality rates were generally low and similar across strategies, with one long-term observational CABG study suggesting reduced death or myocardial infarction with physiology guidance. Graft patency and short-term surgical outcomes were comparable, supporting the safety of deferring physiologically non-significant lesions. In CCS, physiology-guided revascularization improves early PCI outcomes and safely reduces unnecessary interventions, while providing comparable long-term clinical, mortality, and surgical outcomes to angiography-guided strategies.

## Introduction and background

For the anatomical evaluation of coronary artery disease (CAD) affecting the epicardial coronary arteries, invasive coronary angiography is regarded as the gold standard. Chronic coronary syndrome (CCS) is defined according to the contemporary European Society of Cardiology (ESC) guidelines as stable CAD without acute coronary syndromes, including patients with stable angina, ischemia with non-obstructive coronary arteries, or asymptomatic ischemia identified during evaluation for CAD [1]. However, there is often a discrepancy between angiographic appearance and physiological significance when relying solely on visual estimation to determine the functional severity of coronary stenoses, especially in patients with intermediate lesions and CCS [2]. These differences occur because coronary physiology provides a more thorough evaluation of ischemia-producing lesions by integrating the dynamic interaction between coronary blood flow and microvascular resistance, in contrast to angiography.

When compared to angiography-guided techniques, there is mounting evidence that physiology-guided coronary revascularization, which uses hyperemia-based indices such as fractional flow reserve (FFR) and non-hyperemic pressure-derived indices such as instantaneous wave-free ratio (iFR), as well as angiography-derived indices such as quantitative flow ratio (QFR), is more cost-effective and clinically beneficial, especially in the context of percutaneous coronary intervention (PCI) [3-5]. Among these indices, FFR has the most robust outcome-based validation, while iFR has demonstrated non-inferiority to FFR in large randomized trials, and QFR represents an emerging technology with growing but comparatively limited outcome data [6]. In patients with CCS, physiology-guided PCI strategies have been shown to enhance short- and intermediate-term clinical outcomes while reducing unnecessary interventions. However, the role of physiology-guided decision-making outside of PCI remains less clearly defined, particularly when contrasted directly with angiography-guided approaches in other revascularization modalities.

Although PCI and coronary artery bypass grafting (CABG) represent fundamentally different revascularization strategies, they share a common challenge in multivessel CCS: the risk of treating anatomically apparent but functionally non-significant lesions [7]. In PCI, physiological assessment is primarily used to determine whether a stenosis should be treated with stenting, thereby avoiding unnecessary device implantation. In CABG, physiology-guided decision-making is applied differently, aiming to inform graft selection and target vessels by identifying lesions that are likely to generate ischemia and benefit from bypass, while potentially deferring grafting of functionally insignificant stenoses [8]. Thus, despite mechanistic differences, physiology-guided strategies in both modalities seek to optimize revascularization by aligning treatment with ischemic burden rather than angiographic severity alone.

To simplify revascularization and avoid treating functionally inconsequential stenoses, cardiac surgeons and interventional cardiologists are increasingly incorporating physiological lesion assessment into revascularization planning for multivessel CCS [9]. However, contemporary recommendations comparing CABG, PCI, and medical therapy remain largely derived from trials based on anatomical criteria rather than functional lesion assessment, highlighting the need for a comprehensive synthesis of physiology-guided versus angiography-guided approaches across revascularization modalities [9-13].

Previous reviews have evaluated physiology-guided revascularization in selected settings. One review suggested that physiology-guided CABG may reduce all-cause mortality, while myocardial infarction (MI), repeat revascularization, and major adverse cardiovascular events (MACE) remained similar; however, the evidence was limited by small sample sizes and short follow-up durations [14]. Similarly, PCI-focused meta-analyses have demonstrated that physiology-guided PCI reduces early MACE and periprocedural MI by avoiding unnecessary interventions, without a consistent long-term mortality benefit [15]. Importantly, these prior analyses were not restricted to CCS, frequently included heterogeneous clinical presentations (including acute coronary syndromes), evaluated PCI or CABG in isolation, and did not provide an integrated comparison of physiology-guided versus angiography-guided strategies across revascularization modalities in stable disease. Therefore, the present systematic review aims to comprehensively compare physiology-guided versus angiography-guided coronary revascularization in patients with ESC-defined CCS, integrating evidence across both percutaneous and surgical revascularization strategies while accounting for differences in physiological indices and clinical application.

## Review

Methodology

Study Design

This systematic review was reported in accordance with the Preferred Reporting Items for Systematic Reviews and Meta-Analyses (PRISMA) 2020 guidelines [16] to evaluate the comparative effectiveness of physiology-guided coronary revascularization versus angiography-guided strategies in patients with CCS. The review protocol was not prospectively registered in the International Prospective Register of Systematic Reviews (PROSPERO) or Open Science Framework (OSF), and this is acknowledged as a limitation.

Search Strategy

A comprehensive and systematic literature search was performed in PubMed, ScienceDirect, and the Cochrane Library from database inception to December 2025. The search strategy combined controlled vocabulary (MeSH/Emtree terms) and free-text keywords related to fractional flow reserve, quantitative flow ratio, physiology-guided revascularization, angiography-guided revascularization, percutaneous coronary intervention, coronary artery bypass grafting, and chronic coronary syndromes. Reference lists of relevant reviews and eligible articles were manually screened to identify additional studies. Language restrictions were applied a priori, and only studies published in English were included.

Search string used for PubMed: (("Physiology-Guided"[Title/Abstract] OR "physiologic guidance"[Title/Abstract] OR "fractional flow reserve"[Title/Abstract] OR FFR[Title/Abstract] OR "instantaneous wave-free ratio"[Title/Abstract] OR iFR[Title/Abstract] OR "coronary physiology"[Title/Abstract]) AND ("coronary revascularization"[Title/Abstract] OR "percutaneous coronary intervention"[Title/Abstract] OR PCI[Title/Abstract] OR "coronary artery bypass"[Title/Abstract]) AND ("angiography-guided"[Title/Abstract] OR angiography[Title/Abstract]) AND ("chronic coronary syndrome"[Title/Abstract] OR "stable coronary artery disease"[Title/Abstract] OR "stable ischemic heart disease"[Title/Abstract])).

Search string used for ScienceDirect: ("fractional flow reserve" OR FFR OR iFR) AND ("coronary revascularization" OR PCI) AND ("stable coronary artery disease").

Search string used for Cochrane Library: ("fractional flow reserve" OR FFR OR iFR) AND ("percutaneous coronary intervention" OR PCI) AND (stable OR chronic).

Eligibility Criteria

Studies were included in this review if they enrolled adult patients with CCS or stable CAD and evaluated physiology-guided revascularization using invasive measures such as FFR or iFR, or non-invasive/wire-free indices like QFR. Studies enrolling mixed populations were included if the majority of participants had stable presentations consistent with ESC-defined CCS, or if results for stable patients were reported separately; trials focused exclusively on acute coronary syndromes were excluded. Eligible studies compared these physiology-guided strategies with conventional angiography-guided revascularization and reported cardiovascular outcomes, including mortality, MI, repeat revascularization, MACE, or graft- and stent-related outcomes. Study designs considered were randomized controlled trials (RCTs), prospective or retrospective cohort studies, and propensity-matched observational studies. Studies were excluded if they focused exclusively on acute ST-elevation MI, non-coronary interventions, non-comparative designs, case reports, editorials, or conference abstracts without full data. Non-English-language studies were excluded; this may introduce language bias and is acknowledged in the limitations.

Study Selection

Two reviewers independently screened titles and abstracts for relevance. Full texts of potentially eligible studies were then assessed against the inclusion and exclusion criteria. Discrepancies were resolved through discussion and consensus.

Data Extraction

Data were independently extracted using a standardized data collection form. Extracted variables included study characteristics (author, year, design), population details, intervention and comparator strategies, follow-up duration, cardiovascular outcomes with statistical estimates, additional procedural or clinical outcomes, and key conclusions.

Risk of Bias Assessment

The methodological quality of included RCTs was evaluated using the Cochrane Risk of Bias tool, assessing key domains such as random sequence generation, allocation concealment, blinding, completeness of outcome data, and selective reporting. Observational studies were appraised using the Newcastle-Ottawa Scale, which examines participant selection, comparability of study groups, and outcome assessment. Based on predefined thresholds, studies were classified as having low, moderate, or high risk of bias, and these assessments were considered in the interpretation of study findings. It was not used to exclude studies; instead, they informed the interpretation of findings and the strength of conclusions, with greater emphasis placed on evidence from studies judged to have low risk of bias.

Data Synthesis

Data synthesis was performed using a qualitative narrative approach, as substantial clinical and methodological heterogeneity precluded formal meta-analysis. Heterogeneity arose from differences in study design (randomized trials versus observational cohorts), revascularization modality (PCI versus CABG), physiological assessment tools (FFR, iFR, QFR), comparator strategies, follow-up duration, and outcome definitions. To support transparency, a structured heterogeneity table summarizing differences in study populations, physiological indices, cut-off values, and outcome definitions was constructed.

Extracted data were grouped and synthesized according to revascularization strategy (PCI or CABG) and clinical outcome domains, including MACE, all-cause and cardiovascular mortality, MI, repeat or target vessel revascularization, and graft or procedural outcomes. Where available, results were summarized using reported effect estimates, event rates, hazard ratios, risk ratios, and corresponding P-values to allow comparison across studies. The summary statistics table was used to systematically present key outcomes across studies, including death, MI, target vessel revascularization, and composite endpoints, alongside other relevant procedural and surgical outcomes.

Results

**Figure 1:**
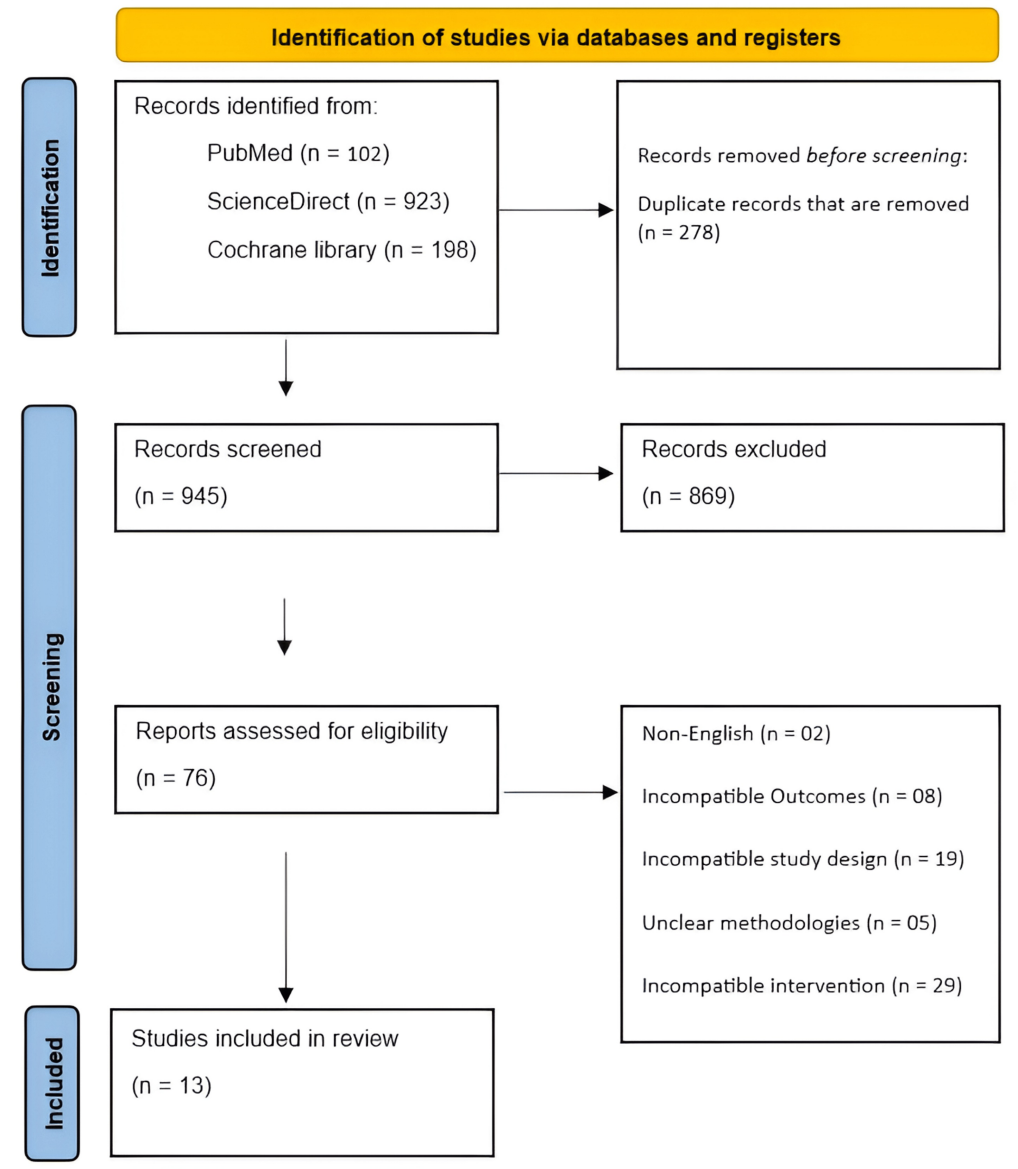
PRISMA flow diagram. PRISMA: Preferred Reporting Items for Systematic Reviews and Meta-Analyses.

A total of 1,223 records were identified through database searching, including PubMed (n = 102), ScienceDirect (n = 923), and the Cochrane Library (n = 198). Of the 945 records screened at the title and abstract level, 869 were excluded primarily due to irrelevance to physiology-guided revascularization in CCS, non-comparative design, non-coronary focus, or clearly ineligible populations, prior to full-text eligibility assessment. The full texts of 76 articles were subsequently assessed for eligibility. Of these, 63 studies were excluded for reasons including non-English language (n = 2), incompatible outcomes (n = 8), incompatible study design (n = 19), unclear methodologies (n = 5), and incompatible interventions (n = 29). Ultimately, 13 studies met the predefined inclusion criteria and were included in the final systematic review (Figure 1).

Study Characteristics

A total of 13 studies were included, comprising multicenter RCTs, single-center RCTs, and retrospective cohort studies. The populations ranged from patients with multivessel CAD undergoing PCI or CABG to patients with intermediate or bifurcation lesions. Sample sizes varied from 100 to 1,500 participants, with follow-up durations ranging from six months to six years. Interventions included physiology-guided PCI or CABG using FFR or iFR, physiology-guided lesion assessment using angiography-derived QFR, and physiology-guided provisional stenting, compared with angiography-guided strategies. Most studies utilized drug-eluting stents for PCI, and physiology-guided CABG involved deferring grafting of lesions with FFR >0.80 (Table 1).

**Table 1:** Characteristics and conclusion of the studies included. PCI: percutaneous coronary intervention; FFR: fractional flow reserve; MI: myocardial infarction; MACE: major adverse cardiovascular events; CABG: coronary artery bypass grafting; iFR: instantaneous wave-free ratio; LAD: left anterior descending artery; QFR: quantitative flow ratio; DES: drug-eluting stent; SB: side branch.

Author (Study)	Year	Study design	Population type	Population number	Intervention	Control	Follow-up time	Key conclusions
Tonino et al. (FAME) [17]	2009	Multicenter randomized controlled trial	Patients ≥18 years with multivessel coronary artery disease undergoing PCI	N = 1005 (angiography-guided PCI, n = 496; FFR-guided PCI, n = 509)	FFR-guided PCI with drug-eluting stents; lesions stented only if FFR ≤0.80	Angiography-guided PCI; all angiographically significant lesions stented	1 year	FFR-guided PCI reduced death, MI, and repeat revascularization at 1 year with fewer stents than angiography-guided PCI
Pijls et al. (FAME 2-year follow-up) [18]	2010	Multicenter randomized controlled trial (extended follow-up)	Patients with multivessel coronary artery disease undergoing PCI	N = 1005 (same cohort as original FAME)	FFR-guided PCI with drug-eluting stents; PCI only if FFR ≤0.80	Angiography-guided PCI	2 years	Benefits of FFR-guided PCI persisted at 2 years, lowering death or MI, confirming the safety of deferring non-significant lesions
van Nunen et al. (FAME 5-year follow-up) [19]	2015	Multicenter randomized controlled trial (long-term follow-up)	Patients with multivessel coronary artery disease	N = 1005 (angiography-guided, n = 496; FFR-guided, n = 509)	FFR-guided PCI; revascularization only if FFR ≤0.80	Angiography-guided PCI	5 years	Long-term safety of FFR-guided PCI confirmed; similar MACE at 5 years with fewer stents and less resource use
Fearon et al. (FAME 3) [20]	2025	Multicenter, open-label, randomized controlled trial	Patients ≥21 years with three-vessel coronary artery disease without left main involvement	N = 1500 (PCI, n=757; CABG, n=743)	FFR-guided PCI using contemporary zotarolimus-eluting stents	Coronary artery bypass grafting	5 years	FFR-guided PCI vs. CABG showed similar death, stroke, or MI at 5 years; PCI had more MI and repeat revascularization, supporting shared decision-making
Thuesen et al. [21]	2018	Single-center randomized controlled trial	Patients referred for coronary artery bypass grafting	N = 100 (FFR-guided CABG, n = 50; angiography-guided CABG, n=50)	FFR-guided CABG with deferral of lesions with FFR >0.80	Angiography-guided CABG (surgeon blinded to FFR)	6 months	FFR-guided CABG had similar short-term graft outcomes as angiography-guided CABG, though deferred lesions showed physiological decline
Fournier et al. [22]	2018	Retrospective cohort with propensity-score matching	Patients undergoing CABG (2006–2010)	N = 627 overall; matched cohort n = 396 (FFR-guided, n = 198; angiography-guided, n = 198)	FFR-guided CABG (≥1 lesion grafted based on FFR)	Angiography-guided CABG (all stenoses grafted)	Median = 85 months (~6 years)	FFR-guided CABG reduced long-term death or MI compared with angiography-guided CABG, suggesting the benefit of physiology-guided surgical revascularization
Moscona et al. [23]	2018	Retrospective cohort study	Patients with moderate coronary lesions (40–70% stenosis) referred for CABG	N = 109 (FFR/iFR-guided vs. angiography-guided CABG)	CABG guided by physiologic assessment (FFR/iFR)	Angiography-guided CABG	18 months	Physiologic assessment can guide more complete and anatomically tailored revascularization in CABG, with trends toward fewer ischemic symptoms and MACEs
Toth et al. (GRAFFITI trial) [24]	2019	Prospective, multicenter, single-blinded randomized controlled trial	Patients undergoing CABG with LAD or left main disease plus ≥1 additional vessel with intermediate stenosis	N = 172 (FFR-guided, n = 88; angiography-guided, n = 84)	FFR-guided CABG (grafting only lesions with FFR ≤0.80)	Angiography-guided CABG	12 months	FFR guidance simplifies CABG surgery without compromising graft patency or short-term clinical outcomes
Sukahri et al. [25]	2024	Single-center pilot observational study	Patients with stable coronary artery disease undergoing coronary angiography/PCI	Screened, N = 770; PCI with QFR vs. PCI without QFR (exact group Ns not specified)	QFR-guided decision-making before and after PCI (QFR <0.80 significant; post-PCI QFR >0.90 acceptable)	Angiography-guided PCI without QFR	6 months	QFR significantly influenced PCI decision-making, particularly pre-intervention, with a trend toward improved short-term outcomes
Biscaglia et al. (AQVA Trial) [26]	2023	Randomized, controlled, parallel-group trial	Patients undergoing PCI (study vessels analysis)	N = 300 patients; 356 vessels (QFR-virtual PCI vs. angiography-guided PCI)	QFR-based virtual PCI planning to optimize post-PCI physiology	Conventional angiography-guided PCI	In-hospital/post-PCI assessment	QFR-based virtual PCI improves the achievement of optimal post-PCI physiological results compared with angiography alone
Rioufol et al. (FUTURE Trial) [27]	2021	Prospective, randomized, open-label superiority trial (stopped early)	Patients with multivessel coronary artery disease	N = 927	FFR-guided treatment strategy for all ≥50% stenoses (revascularization if FFR ≤0.80)	Traditional strategy without systematic FFR	1 year (extended to 24 months)	Routine FFR-guided strategy did not reduce ischemic events or mortality compared with conventional management in multivessel CAD
Park et al. (DEFER-DES Trial) [28]	2015	Randomized controlled trial	Patients with angiographically intermediate coronary artery stenosis	N = 229 (FFR-guided, n = 114; routine-DES, n = 115)	FFR-guided strategy: DES if FFR <0.75; deferral if FFR ≥0.75	Routine DES implantation without FFR	5 years	FFR-guided DES implantation is at least as effective as routine DES and safely avoids unnecessary stent placement
Chen et al. (DKCRUSH-VI Trial) [29]	2015	Multicenter randomized controlled trial	Patients with true coronary bifurcation lesions (Medina 1, 1, 1 or 0, 1, 1)	N = 320 (angio-guided, n = 160; FFR-guided, n = 160)	FFR-guided provisional SB stenting (SB stent if FFR <0.80)	Angiography-guided provisional SB stenting	1 year	FFR guidance reduced SB stenting rates without improving or worsening 1-year clinical outcomes

Quality Assessment

**Figure 2:**
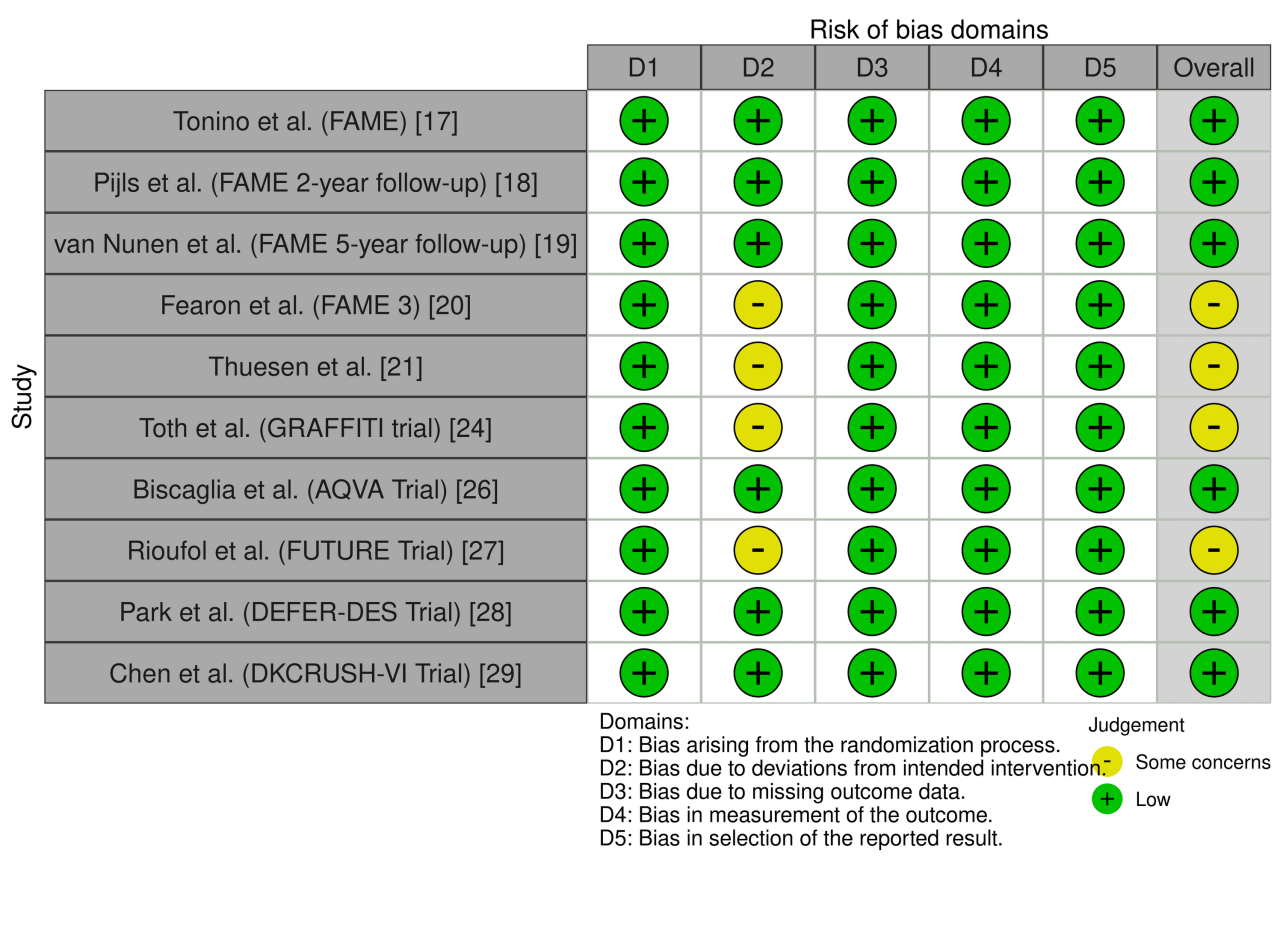
Quality assessment of randomized controlled trials by Risk of Bias 2.0 (RoB 2.0).

Quality assessment of the included studies demonstrated generally robust methodological rigor. Among RCTs, most studies, including FAME [17], FAME 2-year [18], FAME 5-year [19], DEFER-DES [28], DKCRUSH-VI [29], and AQVA [26], were rated as low risk of bias across all Risk of Bias 2 (RoB 2) domains, while FAME 3 [20], Thuesen et al. [21], GRAFFITI [24], and FUTURE [27] showed some concerns due to open-label designs, single-blinding, or early termination (Figure 2). Studies were categorized as low risk of bias if all domains were rated as “low risk,” as having some concerns if at least one domain was rated as “some concerns,” and as high risk if one or more domains were rated as “high risk.” Observational studies assessed using the Newcastle-Ottawa Scale showed low risk for Fournier et al. [22] (score 9), whereas Moscona et al. [23] and Sukahri et al. [25] had moderate risk (score 6) due to lower comparability and outcome assessment scores. Scores of 7-9 were classified as low risk of bias, scores of 5-6 as moderate risk, and scores ≤4 as high risk (Table 2).

**Table 2:** Quality assessment of cohort studies by the Newcastle–Ottawa Scale.

Study	Selection (0–4)	Comparability (0–2)	Outcome (0–3)	Total score (0–9)	Risk of bias
Fournier et al. [22]	4	2	3	9	Low risk
Moscona et al. [23]	3	1	2	6	Moderate risk
Sukahri et al. [25]	3	1	2	6	Moderate risk

Physiology-Guided Lesion Selection and Deferral (FFR/iFR/QFR)

Studies evaluating lesion selection or deferral based on physiological significance were analyzed separately from studies focusing on post-PCI physiological optimization, as these address distinct clinical decision-making questions.

Major Adverse Cardiovascular Events (MACE)

MACE were defined according to individual study protocols and most commonly comprised a composite of death, MI, and repeat or target vessel revascularization. In some trials, additional components such as stroke were included. Given this variability, MACE results are reported using study-specific definitions rather than a single pooled operational definition. Across PCI-focused randomized trials, physiology-guided revascularization was associated with lower early MACE rates and comparable long-term outcomes relative to angiography-guided strategies. Tonino et al. in the landmark FAME trial reported that FFR-guided PCI significantly reduced the composite endpoint of death, MI, or repeat revascularization at one year compared with angiography-guided PCI (13.2% vs. 18.3%, P = 0.02) [17]. Pijls et al. demonstrated that at two years, MACE remained numerically lower with FFR guidance (17.9% vs. 22.4%), although this difference did not reach statistical significance (P = 0.08) [18]. van Nunen et al. reported that by five-year follow-up, MACE rates converged, with no statistically significant difference between strategies (28% vs. 31%, P = 0.31) [19].

In comparisons involving surgical revascularization, composite ischemic outcomes were largely similar between physiology-guided and angiography-guided approaches. Fearon et al. in FAME 3 reported that the composite of death, stroke, or MI at five years did not differ significantly between FFR-guided PCI and CABG (16% vs. 14%; HR: 1.16, P = 0.27) [20]. Thuesen et al. reported no difference in MACE at six months (12% vs. 12%, P = 0.97) [21], and Toth et al. reported no significant difference in composite endpoints at 12 months in the GRAFFITI trial [24]. Observational data from Fournier et al. showed a numerically lower MACE rate with FFR-guided CABG (21% vs. 26%), although this was not statistically significant after adjustment (HR: 0.77, P = 0.21) [22]. Similar non-significant differences were reported in smaller observational cohorts [23] and in trials evaluating routine FFR strategies in multivessel disease (Table 3) [27].

**Table 3:** Statistical results of cardiovascular and other outcomes between physiology-guided coronary revascularization versus angiography. PCI: percutaneous coronary intervention; FFR: fractional flow reserve; MI: myocardial infarction; MACE: major adverse cardiovascular events; CABG: coronary artery bypass grafting; QFR: quantitative flow ratio; SB: side branch; CCS: chronic coronary syndrome; VOCE: vessel-oriented composite endpoint.

Author (Study)	Year	Death	Myocardial infarction (MI)	Target vessel revascularization (TVR)	Major adverse cardiovascular events (MACE)	Other outcomes (with statistics)
Tonino et al. (FAME) [17]	2009	1.8 % vs. 3%	5.7% vs. 8.7%	6.5% vs. 9.5%	13.2% (FFR) vs. 18.3% (Angio), P=0.02	Mean stents/patient: 1.9±1.3 vs. 2.7±1.2, P<0.001; Angina-free at 1 yr: 81% vs. 78% (P=0.20)
Pijls et al. (FAME 2-year follow-up) [18]	2010	Death/MI: 8.4% vs. 12.9%, P=0.02	Included with death	10.6% vs. 12.7%, P=0.30	17.9% vs. 22.4%, P=0.08	Deferred lesions (FFR>0.80): MI 0.2%, revascularization 3.2% at 2 yrs
van Nunen et al. (FAME 5-year follow-up) [19]	2015	All-cause death: 10% (49/496) vs. 9% (44/509), P=0.50	60 vs. 49 events	101 vs. 92 events	31% vs. 28%, P=0.31	Cardiac death: 6% vs. 4%, P=0.26; Death or MI: 20% vs. 17%, P=0.24; Events/patient: 0.42 vs. 0.36, P=0.28
Fearon et al. (FAME 3) [20]	2025	7% vs. 7%; HR: 0.99 (0.67–1.46)	8% vs. 5%; HR: 1.57 (1.04–2.36)	16% vs. 8%; HR: 2.02 (1.46–2.79)	Death/Stroke/MI: 16% vs. 14%; HR: 1.16 (0.89–1.52), P=0.27	Higher repeat revascularization with PCI; 5-yr follow-up completeness >94%
Thuesen et al. [21]	2018	0% vs. 4%, P=0.24	2% vs. 0%, P=0.50	6% vs. 6%, P=1.00	12% vs. 12%, P=0.97	Graft failure per patient: 16 vs. 12, P=0.97; Graft occlusion: 15% vs. 15%; CCS III–IV angina: 10% vs. 4%, P=0.29
Fournier et al. [22]	2018	Death or MI: 16% (FFR) vs. 25% (Angio); HR: 0.59 (0.38–0.93), P=0.020	Included with death	Included in composite	21% vs. 26%; HR: 0.77 (0.51–1.16), P=0.21	Fewer graft anastomoses; lower on-pump CABG rates in the FFR group
Moscona et al. [23]	2018	5.3% (5/95) vs. 7.1% (1/14), P=0.401	2.1% (2/95) vs. 0%, P=0.759	4.2% (4/95) vs. 0%, P=0.572	11.6% vs. 7.1%, P=0.369	Mean time to MACE: 16.7 vs. 17.3 months, P=0.609; Angina: 6.3% vs. 0%, P=0.429
Toth et al. (GRAFFITI trial) [24]	2019	In-hospital: 0% vs. 1.1%, P=0.327	In-hospital: 2.4% vs. 0%, P=0.145	In-hospital: 2.4% vs. 0%, P=0.145	No significant difference (death/MI/stroke/TVR composite)	Hospital stay: 11 vs. 11 days, P=0.367; 1-yr graft patency: 80% vs. 81%, P=0.885; Anastomoses: 2 vs. 3, P=0.004
Sukahri et al. [25]	2024	0 vs. 1 cardiac death, P=0.316	0 vs. 0	0 vs. 0	No significant difference in VOCE	PCI decision change: pre-PCI = 39%, post-PCI = 22%
Biscaglia et al. (AQVA Trial) [26]	2023	Not reported	Not reported	Not reported	Not the primary endpoint	Suboptimal post-PCI QFR <0.90: 6.6% vs. 15.1%, P=0.009; Procedure duration longer (P=0.06); Stent length trend lower (P=0.08)
Rioufol et al. (FUTURE Trial) [27]	2021	3.7% vs. 1.5%; HR: 2.34 (0.97–5.68), P=0.06	6.1% vs. 6.0%; HR: 1.03 (0.61–1.74), P=0.90	8.0% vs. 9.9%; HR: 0.79 (0.51–1.22), P=0.28	14.6% vs. 14.4%; HR: 0.97 (0.69–1.36), P=0.85	Stroke: 0.2% vs. 1.5%, P=0.06; FFR reduced revascularization, more medical therapy
Park et al. (DEFER-DES Trial) [28]	2015	Cardiac death or MI: 3.5% vs. 6.1%, P=0.54	Included with death	8.8% vs. 7.8%, P=0.82	11.4% vs. 13.9%, P=0.69	Only ~25% lesions functionally significant; avoided unnecessary stenting
Chen et al. (DKCRUSH-VI Trial) [29]	2015	0% vs. 0%	12.5% vs. 11.9%, P=0.86	0% vs. 0%	12.5% vs. 11.9%, P=0.86	SB stenting attempted less with FFR: 25.9% vs. 38.1%, P=0.01

All-Cause and Cardiovascular Mortality

No consistent mortality benefit was demonstrated with physiology-guided revascularization. In PCI-based trials, mortality rates were low and comparable between strategies across follow-up durations. van Nunen et al. reported that at five years in FAME, all-cause mortality did not differ between FFR-guided and angiography-guided PCI (10% vs. 9%, P = 0.50), nor did cardiac mortality (6% vs. 4%, P = 0.26) [19]. Fearon et al. reported that in FAME 3, five-year all-cause mortality was identical between FFR-guided PCI and CABG (7% vs. 7%; HR: 0.99) [20].

In surgical cohorts, early mortality remained low and statistically similar between strategies, including at six months (0% vs. 4%, P = 0.24) [21] and during in-hospital follow-up (0% vs. 1.1%, P = 0.327) [24]. Rioufol et al. in the FUTURE trial reported numerically higher all-cause mortality in the FFR-guided group at one year (3.7% vs. 1.5%) [27], though this difference did not reach statistical significance (HR: 2.34, P = 0.06). In contrast, long-term observational data from Fournier et al. suggested a significant reduction in the composite of death or MI with FFR-guided CABG (16% vs. 25%; HR: 0.59, P = 0.020) (Table 3) [22].

Myocardial Infarction

MI rates were generally similar between physiology-guided and angiography-guided strategies, particularly in CABG populations. In PCI cohorts, MI was frequently reported within composite outcomes. Tonino et al. did not report MI separately in FAME [17], whereas Pijls et al. demonstrated a significant reduction in the combined endpoint of death or MI at two years with FFR guidance (8.4% vs. 12.9%, P = 0.02). van Nunen et al. reported that at five years, MI event counts were numerically lower in the FFR group but not statistically compared (60 vs. 49 events) [19].

Fearon et al. reported that in FAME 3, MI occurred more frequently in the PCI group than in the CABG group despite physiology guidance (8% vs. 5%; HR: 1.57, P = 0.02) [20]. CABG-focused trials demonstrated low and comparable MI rates at short-term follow-up [21,24]. Observational studies similarly reported no significant differences in MI between strategies (Table 3) [23,27-29].

Target Vessel Revascularization and Repeat Revascularization

Physiology-guided strategies were associated with similar or numerically lower rates of repeat revascularization in most PCI trials. Pijls et al. reported that in FAME 2, repeat revascularization was numerically lower with FFR-guided PCI (10.6% vs. 12.7%), although this difference was not statistically significant (P = 0.30) [18]. van Nunen et al. demonstrated that long-term follow-up showed comparable revascularization events between groups (101 vs. 92) [19].

In contrast, Fearon et al. reported that FAME 3 showed significantly higher repeat revascularization with FFR-guided PCI compared with CABG (16% vs. 8%; HR: 2.02, P < 0.001) [20]. In CABG-only studies, revascularization rates were low and similar between groups at both short- and intermediate-term follow-up [21,24]. Observational cohorts reported no statistically significant differences in target vessel revascularization (Table 3) [23,27].

Graft Patency and Surgical Outcomes

Graft patency outcomes were similar between physiology-guided and angiography-guided CABG strategies. Thuesen et al. reported that at six months, graft failure rates per patient did not differ significantly (16 vs. 12, P = 0.97), and graft occlusion rates were identical (15% vs. 15%) [21]. Toth et al. reported that at 12 months, overall graft patency was comparable between groups (80% vs. 81%, P = 0.885) [24]. These findings demonstrated comparable graft patency and graft occlusion rates between physiology-guided and angiography-guided CABG strategies at short-term follow-up (Table 3).

Post-PCI Physiological Optimization (QFR-Based Virtual PCI Planning)

Studies evaluating post-PCI physiological optimization addressed a distinct clinical question focused on improving final physiological results rather than lesion selection or deferral. In the AQVA trial, QFR-based virtual PCI planning was associated with fewer vessels exhibiting suboptimal post-PCI physiology (QFR <0.90) compared with angiography-guided PCI (6.6% vs. 15.1%, P = 0.009) [23]. These studies primarily reported procedural and physiological endpoints rather than long-term clinical outcomes.

Clinical Heterogeneity

Clinical heterogeneity was substantial across studies with respect to lesion type (multivessel vs. intermediate vs. bifurcation), modality (PCI vs. CABG), physiological indices (FFR/iFR/QFR), and thresholds (FFR ≤0.80 vs. <0.75; QFR <0.80; post-PCI QFR >0.90), and outcome definitions (clinical MACE vs. graft patency vs. physiologic post-PCI endpoints) (Table 4).

**Table 4:** Structured heterogeneity table (population, physiology indices/cut-offs, and outcome definitions). PCI: percutaneous coronary intervention; CAD: coronary artery disease; FFR: fractional flow reserve; MI: myocardial infarction; MACE: major adverse cardiovascular events; CABG: coronary artery bypass grafting; iFR: instantaneous wave-free ratio; LAD: left anterior descending artery; TVR: target vessel revascularization; QFR: quantitative flow ratio; VOCE: vessel-oriented composite endpoint; DES: drug-eluting stent; SB: side branch.

Author (Study)	Revascularization setting	Study population (key differences)	Physiology index used	Cut-off/target used for decision-making	Comparator strategy	Main outcome definition(s) reported/emphasized (heterogeneity)
Tonino et al. (FAME) [17]	PCI	Multivessel CAD; ≥18 years; angiographically significant lesions	FFR	FFR ≤0.80 → stent; >0.80 deferred	Angiography-guided PCI (all significant lesions stented)	MACE composite: death/MI/repeat revascularization (primary composite at 1 year)
Pijls et al. (FAME 2-year follow-up) [18]	PCI	Same FAME cohort; extended follow-up	FFR	FFR ≤0.80 → PCI; >0.80 deferred	Angiography-guided PCI	MACE composite (death/MI/repeat revascularization), plus deferred-lesion outcomes (MI and revascularization in lesions with FFR >0.80)
van Nunen et al. (FAME 5-year follow-up) [19]	PCI	Same FAME cohort; long-term follow-up	FFR	FFR ≤0.80 → PCI; >0.80 deferred	Angiography-guided PCI	Long-term MACE (study-defined composite), plus death (all-cause/cardiac) and event counts; composite definitions/timepoints differ from 1 to 2 year reports
Fearon et al. (FAME 3) [20]	Modality comparison (PCI vs. CABG)	Three-vessel CAD, ≥21 years; no left main involvement	FFR (in PCI arm)	FFR-guided PCI (lesions treated per FFR strategy; threshold aligned to contemporary practice)	CABG (not angiography-guided PCI)	Primary composite differs: death/stroke/MI (5 years); also repeat revascularization and MI (PCI higher) — not physiology vs. angiography
Thuesen et al. [21]	CABG	Patients referred for CABG	FFR	FFR >0.80 lesions deferred from grafting	Angiography-guided CABG (surgeon blinded to FFR)	Graft-related outcomes (graft failure/occlusion) + short-term MACE (6 months); CABG outcomes differ from PCI composites
Fournier et al. [22]	CABG	CABG cohort (2006–2010); propensity-matched	FFR	Grafting guided by FFR (≥1 lesion grafted based on physiology)	Angiography-guided CABG (all stenoses grafted)	Composite endpoints vary: death/MI composite; MACE reported as adjusted HR; longer-term follow-up (median ~6 years)
Moscona et al. [23]	CABG	Moderate lesions (40–70% stenosis) referred for CABG	FFR/iFR	Physiology-guided CABG (index-guided decision-making; threshold not uniformly reported)	Angiography-guided CABG	Outcomes include symptoms + MACE (18 months); definitions and small sample cause variability
Toth et al. (GRAFFITI trial) [24]	CABG	LAD or left main + ≥1 additional intermediate stenosis	FFR	FFR ≤0.80 grafted; >0.80 deferred	Angiography-guided CABG	Composite clinical outcomes (death/MI/stroke/TVR composite) + graft patency (1 year); outcome focus differs from PCI trials
Sukahri et al. [25]	PCI	Stable CAD; angiography/PCI workflow	QFR	QFR <0.80 significant; post-PCI QFR >0.90 acceptable	PCI without QFR (angiography-guided)	Outcome reported as VOCE/ short-term clinical trend + procedural decision change; endpoints not identical to classic MACE
Biscaglia et al. (AQVA Trial) [26]	PCI	PCI patients; vessel-level analysis	QFR (virtual PCI planning)	Target: optimize post-PCI physiology; suboptimal post-PCI QFR <0.90	Conventional angiography-guided PCI	Physiologic procedural endpoint (post-PCI QFR optimization) rather than clinical MACE; in-hospital/post-PCI assessment
Rioufol et al. (FUTURE Trial) [27]	Strategy trial (PCI/medical management pathway)	Multivessel CAD; trial stopped early	FFR	FFR ≤0.80 triggers revascularization for ≥50% stenoses	Traditional strategy without systematic FFR	Outcomes include ischemic events/mortality; early stopping + mixed strategy pathways increase heterogeneity in endpoint interpretation
Park et al. (DEFER-DES Trial) [28]	PCI	Intermediate stenosis	FFR	FFR <0.75 → DES; FFR ≥0.75 deferred	Routine DES without FFR	Outcomes: cardiac death/MI composite + MACE and revascularization over long follow-up; different threshold (0.75) vs. most other PCI trials
Chen et al. (DKCRUSH-VI Trial) [29]	PCI (bifurcation)	True bifurcation lesions (Medina 1,1,1 or 0,1,1)	FFR (side-branch)	SB stent if FFR <0.80	Angio-guided provisional SB stenting	Outcomes emphasize procedural strategy (SB stenting rate) with clinical outcomes at 1 year; endpoint structure differs from multivessel PCI trials

Discussion

This systematic review evaluated evidence comparing physiology-guided coronary revascularization with angiography-guided strategies in chronic coronary syndrome. Findings show that FFR- and QFR-guided approaches safely defer non-ischemic lesions, reduce unnecessary interventions, and achieve comparable or improved clinical outcomes. Early landmark trials in PCI established the clinical value of physiology guidance. The FAME trial showed that FFR-guided PCI significantly reduced the composite of death, MI, or repeat revascularization at one year compared with angiography-guided PCI, while using substantially fewer stents [14]. These benefits were sustained at two-year follow-up, with lower rates of death or MI and very low event rates in lesions deferred based on preserved FFR, supporting the long-term safety of deferral [15]. At five years, although MACE were similar between strategies, FFR guidance maintained durable reductions in stent use and resource utilization without late harm [16].

More recent randomized and observational studies further refine these observations. Trials such as DEFER-DES and DKCRUSH-VI demonstrated that FFR guidance safely avoids unnecessary stenting in angiographically intermediate or complex bifurcation lesions, without increasing long-term MACE [25,26]. However, not all studies showed superiority in hard outcomes. The FUTURE trial, which applied a routine FFR-guided strategy across multivessel disease, found no reduction in ischemic events compared with conventional management and raised concerns regarding early mortality imbalance, highlighting that patient selection, operator experience, and treatment strategy context remain critical [24].

The role of physiology guidance has also expanded beyond PCI into surgical revascularization. Randomized and propensity-matched CABG studies suggest that FFR-guided grafting can reduce the number of anastomoses and avoid bypassing non-ischemic vessels, without compromising graft patency or short-term outcomes [18,21]. Importantly, long-term observational data indicate a potential reduction in death or MI with FFR-guided CABG compared with angiography-guided surgery, suggesting that physiological lesion selection may confer a durable prognostic benefit in surgical patients [19].

When physiology-guided PCI was compared directly with CABG in complex disease, as in the recent FAME 3 trial, no significant difference was observed in the composite of death, stroke, or MI at five years. However, PCI was associated with higher rates of MI and repeat revascularization, whereas CABG remained more durable in preventing repeat procedures [17]. These findings underscore that while physiology guidance optimizes PCI outcomes, it does not eliminate the inherent differences between revascularization modalities, reinforcing the importance of heart-team-based, shared decision-making.

Emerging wire-free physiological tools such as QFR show promise in further simplifying functional assessment. Studies, including the AQVA trial, and pilot observational data demonstrate that QFR-guided planning improves post-PCI physiological results and significantly influences treatment decisions, with trends toward fewer suboptimal outcomes [22,23]. While short-term clinical outcomes appear comparable, larger trials with longer follow-up are required before widespread adoption.

Comparison with existing systematic reviews and meta-analyses supports the findings of the present review. A prior CABG-focused meta-analysis showed that physiology-guided surgery was associated with reduced all-cause mortality compared with angiography-guided CABG, while MI, target vessel revascularization, and MACE were similar, aligning with our synthesis and suggesting that physiological lesion selection can safely simplify surgery and may confer a survival benefit despite limitations in sample size and follow-up [11]. Likewise, an individual patient data meta-analysis of randomized PCI trials demonstrated lower one-year major adverse cardiac events and MI with FFR guidance, driven mainly by fewer peri-procedural MIs and reduced stent use, consistent with the PCI evidence in our review and indicating that the primary benefit of physiology-guided PCI is the avoidance of unnecessary intervention and procedural harm rather than long-term mortality reduction [12].

This review is limited by marked heterogeneity in study designs, patient populations, revascularization modalities (PCI vs. CABG), and physiological assessment tools (FFR, iFR, QFR), which constrained direct comparability and precluded formal meta-analysis. Several CABG-focused studies were observational or single-center with small sample sizes, increasing susceptibility to residual confounding and selection bias, while some trials had short follow-up or were terminated early, limiting assessment of long-term prognostic effects. Additionally, evolving stent platforms, surgical techniques, and physiology thresholds over time may affect the generalizability of older trial results to contemporary practice. The review protocol was not prospectively registered (e.g., in PROSPERO), which may increase the risk of selective reporting or methodological bias. Furthermore, the exclusion of non-English language studies introduces the potential for language bias, particularly relevant for surgical observational literature. Future studies should prioritize large, multicenter randomized trials directly comparing physiology-guided and angiography-guided strategies across both PCI and CABG with standardized endpoints. Long-term evaluation of emerging non-invasive physiology indices and their integration into hybrid revascularization strategies is also warranted.

## Conclusions

This systematic review demonstrates that physiology-guided coronary revascularization provides a clinically effective and safe alternative to angiography-guided strategies in patients with CCS. By incorporating functional lesion assessment, physiology-guided approaches reduce unnecessary revascularization and improve early outcomes following PCI, while maintaining comparable long-term rates of MACE, mortality, MI, and repeat revascularization. In surgical settings, deferral of physiologically non-significant lesions does not compromise graft patency or short-term outcomes, supporting the broader applicability of physiology-guided decision-making across revascularization modalities. Overall, these findings support the integration of coronary physiology into routine clinical practice to optimize individualized revascularization strategies in stable CAD.
